# The association between serum testosterone level and congestive heart failure in US male adults: data from National Health and Nutrition Examination Survey (NHANES) 2011–2016

**DOI:** 10.1186/s12958-023-01171-w

**Published:** 2024-01-02

**Authors:** Xiangpeng Zhan, Yang Liu, Tao Chen, Hao Wan, Situ Xiong, Sheng Li, Xinxi Deng, Luyao Chen, Bin Fu

**Affiliations:** 1 Department of Urology, The First Affiliated Hospital, Jiangxi Medical College, Nanchang University, Nanchang, Jiangxi Province China; 2 Department of Cardiology, The Second Affiliated Hospital of Nanchang University, Nanchang, Jiangxi Province China; 3 Department of Urology, Jiu Jiang first People’s Hospital, Jiujiang, Jiangxi Province China

**Keywords:** Testosterone level, Congestive heart failure, NHANES, Association, Prognostic factors

## Abstract

**Background:**

This study aimed to investigate the relationship between serum testosterone levels and the risk of congestive heart failure (CHF) in adult males. Previous research has suggested a potential link between serum testosterone and cardiovascular health, but the findings have been inconclusive.

**Methods:**

This study was cross-sectional, and the data were obtained from the 2011–2016 cycle of the National Health and Nutrition Examination Survey (NHANES), which included a sample of 6,841 male participants. Serum testosterone levels were measured using a standardized assay, and CHF status was assessed through self-reporting. Covariates such as age, ethnicity, lifestyle factors, and health conditions were considered in the analysis.

**Results:**

Among the participants, 242 individuals had a documented history of CHF. We observed a linear correlation between serum testosterone levels and CHF occurrence, with higher serum testosterone levels associated with a decreased risk of CHF (Q4 vs. Q1, OR = 0.29, 95% CI: 0.19–0.47, P < 0.001). After adjusting for confounding variables, multivariate analysis revealed that high serum testosterone levels remained significantly associated with a lower risk of CHF (OR: 0.47, 95% CI: 0.27–0.80, P = 0.01). Subgroup analysis indicated a significant association between high serum testosterone levels and reduced CHF risk in individuals over 50 years old.

**Conclusion:**

Our findings suggest that the serum testosterone level was positively associated with CHF in adult males. This study highlights the potential role of serum testosterone in cardiovascular health, particularly in older individuals. Further research is needed to elucidate the underlying mechanisms and explore the clinical implications of these findings.

## Introduction

Serum testosterone was a crucial hormone primarily secreted by the testes, playing a key role in male reproductive functions [1]. Only a small fraction of testosterone circulated freely in the bloodstream, while the majority bound to proteins such as albumin and sex hormone-binding globulin (SHBG). Serum testosterone levels varied with the diurnal rhythm and fluctuated throughout an individual’s lifespan. As men aged, serum testosterone levels gradually declined, while the circulating levels of SHBG increased. This decline sparked interest in understanding the relationship between serum testosterone levels and various physiological processes, including cardiovascular health [2]. Multiple factors influenced the cardiovascular effects of serum testosterone, including plasma levels, cellular metabolism, intracellular pathway regulation, and androgen receptor expression [3]. Serum testosterone had both anabolic and androgenic effects, significantly impacting the cardiovascular system. The primary mode of serum testosterone action was believed to be mediated by genomic mechanisms through androgen receptors, but emerging evidence suggested the involvement of non-genomic pathways in its rapid effects. These pathways included the rapid induction of traditional second-messenger signaling cascades, such as protein kinase activation and regulation of intracellular calcium levels [4]. Several studies explored the relationship between serum testosterone, male hypogonadism, and the cardiovascular system. Low serum testosterone levels were associated with cardiovascular risks and diseases, major adverse cardiac events, and mortality caused by cardiovascular events [5]. Longitudinal studies showed that untreated hypogonadal patients not only experienced sexual dysfunction but also unfavorable outcomes in metabolic, cardiovascular, skeletal muscle (sarcopenia), and exercise capacity.

Many studies suggested that serum testosterone replacement therapy (TRT) was beneficial for metabolic and cardiovascular risks in hypogonadal men. TRT improved parameters such as fat/lean body mass ratio, waist circumference, blood glucose, insulin resistance, lipid profile, and blood pressure. Additionally, it might have a protective effect on cardiovascular events and mortality in older men with serum testosterone deficiency who were at high risk for such events. The relationship between low serum testosterone levels and chronic heart failure (HF) was extensively investigated. It was found that serum testosterone deficiency was common in male HF patients, and serum testosterone levels were associated with clinical parameters such as ejection fraction, exercise capacity, and New York Heart Association (NYHA) functional class, as well as prognosis, including HF rehospitalization and mortality. While TRT might be beneficial for male HF patients with low androgen levels, it was contraindicated in those with unstable and severe HF [3, 5–7]. However, the consensus on this matter was based on low-quality evidence and lacked definitive data.

Although the bidirectional relationship between hypogonadism and cardiovascular disease was elucidated, the connection between serum testosterone and congestive heart failure remained controversial. Our hypothesis was that sex hormones might provide the physiological basis for observed changes in stone formation and explain known complications affecting CHF. Therefore, the primary objective of our study was to assess the relationship between serum testosterone levels and CHF using data from the National Health and Nutrition Examination Survey (NHANES). Our study, a large-scale cross-sectional survey, aimed to provide new insights into the relationship between serum testosterone levels and CHF in male participants.

## Materials and methods

### Data and sample sources

The study utilized data from the National Health and Nutrition Examination Survey (NHANES), which is conducted by the National Center for Health Statistics (NCHS). NHANES is a comprehensive survey that aims to collect representative information on the health and nutrition of the non-institutionalized civilian population in the United States. To ensure a diverse sample, NHANES uses a stratified, multistage probability approach to select participants from across the country. The survey collects data through standardized in-home interviews, physical examinations, and laboratory tests carried out at mobile examination centers.

**Fig. 1:**
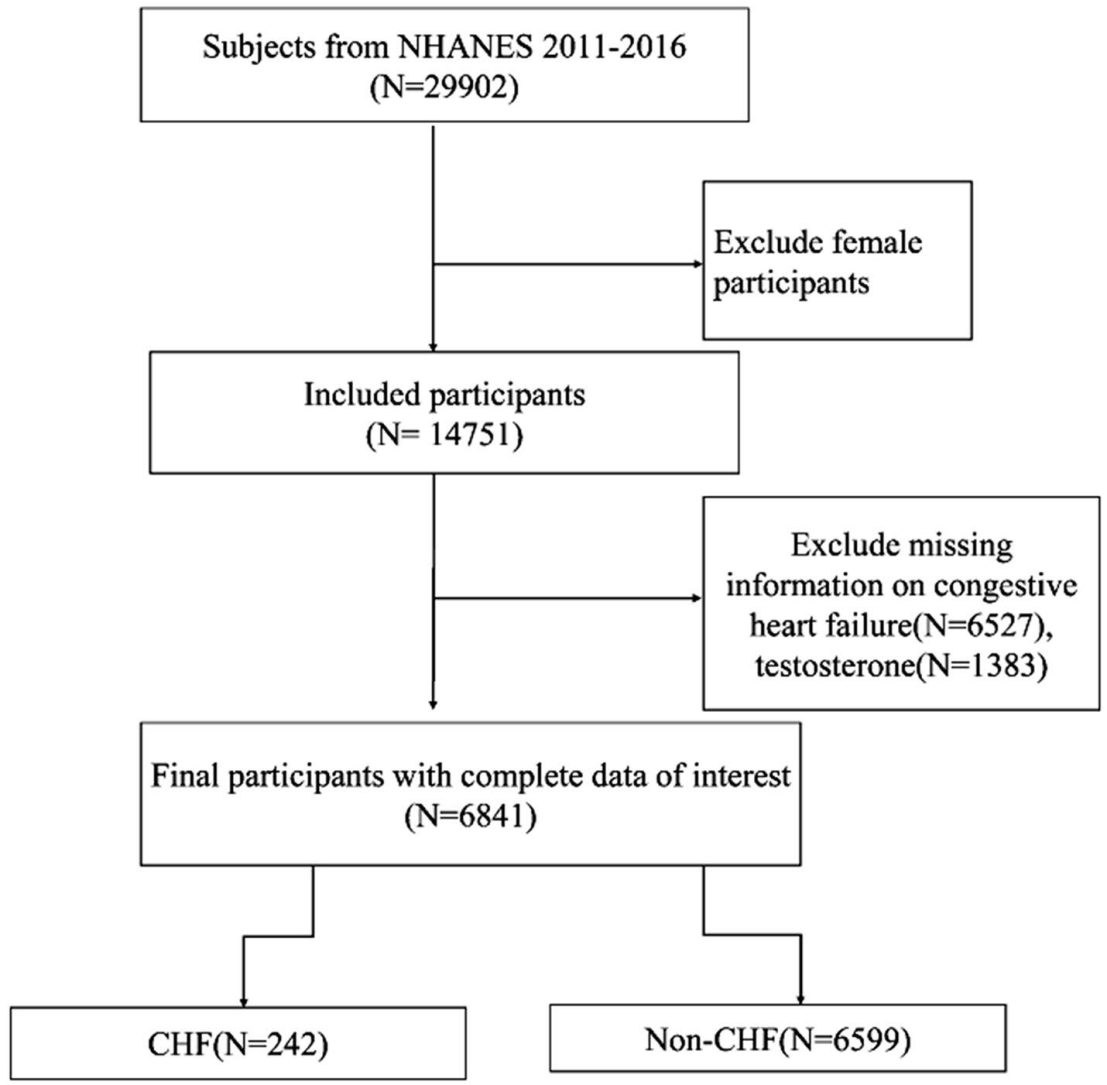
Overview of participants screening. NHANES: National Health and Nutrition Examination Survey, CHF: congestive heart failure

In this particular study, our focus was on adult males from the NHANES 2011–2016 cycle. The initial sample consisted of 28,802 participants. We excluded boys under the age of 20 as clinical androgen decline is uncommon in this age group, and there were not enough participants (n < 10) to conduct robust analysis. Individuals who showed early signs of androgen decline may be due to genetic conditions or exposure to unusually high doses of medication or toxic substances beyond normal levels of everyday exposure. Additionally, we excluded individuals who were missing serum testosterone and congestive heart failure information, resulting in a final analysis sample of 6,841 participants (Fig. 1).

### Assessment of serum testosterone level

After an overnight period without food, blood samples were collected sometime between 8:30 a.m. and 11:30 a.m. These samples were then analyzed for serum testosterone levels using a competitive electrochemiluminescence immunoassay on the 2010 Elecsys autoanalyzer, a device manufactured by Roche Diagnostics in Indianapolis, IN, USA. The assay had a minimum detection limit of 0.02 ng/mL. Serum testosterone and other sex steroid hormones for the current NHANES cycle were analyzed at Boston Children’s Hospital in Boston, MA, USA. The laboratory technicians responsible for the analysis were intentionally blinded to the participants’ characteristics. More information about the NHANES laboratory procedure for serum testosterone determination can be found at the following link: https://wwwn.cdc.gov/Nchs/Nhanes/2013-2014/TST_H.html.

Serum testosterone levels were explored in relation to the occurrence of congestive heart failure (CHF) using serum testosterone levels as both a categorical variable with four categories and as a comparison between low serum testosterone levels(< 300 ng/dl) and normal serum testosterone levels(≥ 300 ng/dl).

### Congestive heart failure assessment

Congestive heart failure is defined in the study by asking participants a specific question regarding their medical history: “Have you ever been diagnosed with congestive heart failure by a doctor or other healthcare professional?” If participants respond affirmatively, their response is recorded as indicating the presence of congestive heart failure.

### Covariates

In order to account for potential confounding factors, the study included several covariates that could have an impact on the relationship between serum testosterone and congestive heart failure (CHF). These covariates encompassed various demographic characteristics of the study population, including age, gender, ethnicity, marital status, educational level, smoking status, alcohol consumption, body mass index (BMI), and annual household income. Additionally, important health risk factors such as diabetes, hypertension, and hyperlipidemia were also considered. A comprehensive classification of these covariates can be found in Table 1.

**Table 1:** Characteristics of the study population by total testosterone(ng/dl)

Variable	Total (N = 6841)	Q1 [1.44,286.75]	Q2 (286.75,384.97]	Q3 (384.97,508]	Q4 (508,2543.99]	P-value
Age (mean, years)	46.98(0.36)	50.16(0.47)	47.49(0.55)	45.46(0.66)	44.94(0.73)	< 0.0001
Ethnic						0.01
black	9.63%	8.67%	8.48%	9.98%	11.35%	
white	67.51%	69.92%	66.42%	67.72%	66.06%	
other	22.86%	21.42	25.10%	22.30%	22.59%	
Marital status						< 0.0001
Married	58.32%	67.42%	62.16%	56.90%	47.14%	
SDW	12.54%	12.60%	11.72%	12.18%	13.67%	
unmarried	29.14%	19.98%	26.12%	30.92%	39.19%	
Annual household income						0.01
0-19.999$	12.11%	11.91%	10.12%	11.95%	14.47%	
20.000-54.999$	34.77%	34.47%	32.45%	34.08%	38.10%	
55.000-74.999$	12.88%	13.36%	14.31%	12.65%	11.23%	
75.000$	40.23%	40.27%	43.13%	41.32%	36.20%	
Education						0.5
High school graduate or under	37.89%	37.57%	37.55%	36.59%	39.88%	
Some college or above	62.11%	62.43%	62.45%	63.41%	60.12%	
Smoke						< 0.0001
former	28.84%	35.21%	28.55%	26.06%	25.80%	
never	49.77%	48.79%	52.35%	51.96%	45.89%	
now	21.39%	15.99%	19.09%	21.98%	28.31%	
Alcohol consumption						< 0.001
No	26.38%	31.94%	26.27%	24.37%	23.18%	
Yes	73.62%	68.06%	73.73%	75.63%	76.82%	
DM						< 0.0001
DM	15.57%	24.21%	16.91%	11.66%	9.88%	
IFG	6.02%	5.22%	5.61%	7.27%	5.91%	
IGT	3.64%	3.82%	4.03%	3.44%	3.26%	
no	74.78%	66.75%	73.45%	77.63%	80.95%	
Hypertension						< 0.0001
no	60.60%	50.43%	58.32%	64.07%	69.17%	
yes	39.40%	49.57%	41.68%	35.93%	30.83%	
Congestive heart failure						< 0.0001
no	97.61%	95.81%	97.45%	98.38%	98.72%	
yes	2.39%	4.19%	2.55%	1.62%	1.28%	
Hyperlipidemia						< 0.0001
no	31.33%	19.10%	26.82%	36.05%	42.84%	
yes	68.67%	80.90%	73.18%	63.95%	57.16%	
BMI (kg/m2)						< 0.0001
<25	26.29%	10.47%	19.15%	28.33%	46.65%	
≥30	36.15%	56.07%	43.46%	29.67%	16.21%	
25-29.9	37.56%	33.46%	37.39%	42.00%	37.14%	

Q: quartile, DM: Diabetes mellitus, IFG: Impaired fasting glucose, IGT: impaired glucose tolerance, SDW: Separated, Divorced, Widowed; BMI: Body mass index

### Statistical analyses

We ensured adherence to the NHANES analytical reporting guidelines, which account for the intricate survey design by assigning weights to participants to correct for oversampling of specific subgroups. For the comparison of continuous variables, we utilized one-way analysis of variance, while chi-square tests were employed for categorical variables. Restricted cubic spline was used to assess linear association between serum testosterone levels and CHF. Multivariate logistic regression analysis was conducted using different models to examine the correlation between serum testosterone levels and risk of CHF.

In Model 1, no adjustments were made for confounding variables. However, in Model 2, adjustments were made for age and race. In Model 3, we further incorporated additional factors, such as marital status, household income, education level, smoking and drinking habits, BMI, hypertension, diabetes, and hyperlipidemia. Furthermore, we performed subgroup analysis based on age (< 50 years, ≥ 50 years) to explore the association between serum testosterone levels and risk of CHF.

The “nhanesR” software package was utilized for data extraction and analysis. To establish statistically significant differences, a significance level of P < 0.05 (two-tailed) was adopted.

## Results

In the NHANES Continuous Surveys conducted between 2011 and 2016, we surveyed a total of 6,841 male participants regarding their history of congestive heart failure (CHF). Out of these 6,841 subjects with comprehensive data, it was discovered that there were 242 cases with a documented history of CHF. Participants with low levels of serum testosterone had a higher proportion of CHF patients (P < 0.0001).

**Fig. 2:**
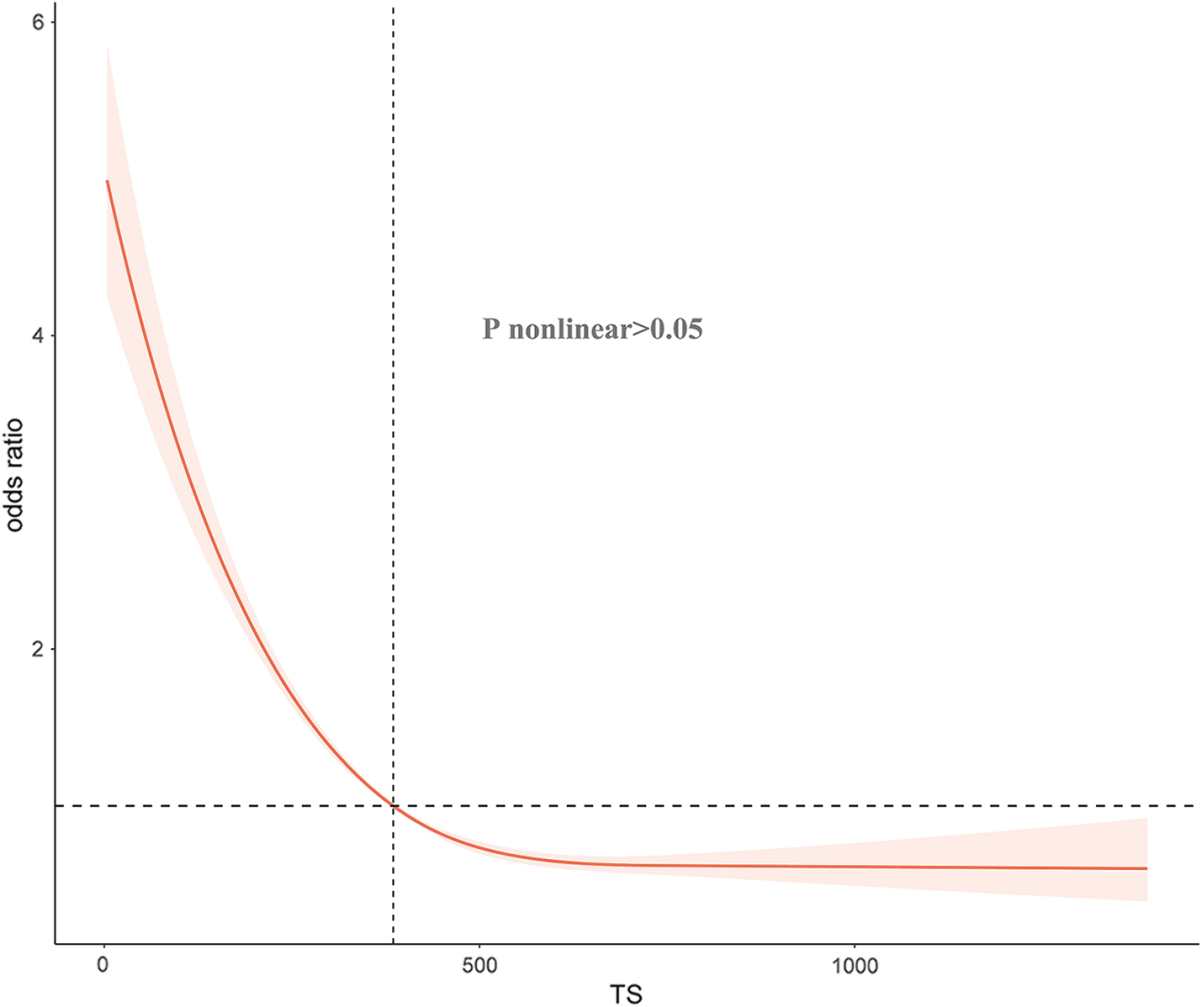
Restricted cubic spline for relation of serum testosterone with risk of congestive heart failure

Overall, the serum testosterone level was positively associated with CHF (nonlinear p > 0.05, Fig. 2). After adjusting for other potential confounding factors, we established three models to assess the independent impact of the overall serum testosterone level on CHF. The univariate analysis showed that a higher level of serum testosterone is a protective factor for CHF (Q4 vs. Q1, OR = 0.29, 95% CI: 0.19–0.47, P < 0.001, Ptrend < 0.001, Table 2). Additionally, individuals with normal serum testosterone levels have a lower proportion of CHF compared to those with low serum testosterone levels (≥ 300 vs. <300, OR = 0.46, 95% CI: 0.33–0.63, P < 0.001, Table 3). After adjusting for age and ethnicity, the model still indicates a significant correlation between high serum testosterone levels and a lower proportion of CHF (OR: 0.34, 95% CI: 0.22–0.54, P < 0.001) when comparing the fourth quartile to the first quartile. Furthermore, after adjusting for age, ethnicity, marital status, annual household income, education, smoking, alcohol consumption, BMI, diabetes, hypertension, and hyperlipidemia, the multivariable logistic regression model shows that high serum testosterone levels are still associated with CHF (OR: 0.47, 95% CI: 0.27–0.80, P = 0.01). Additionally, normal serum testosterone levels group have a significantly lower proportion of developing CHF compared to those with low serum testosterone levels (OR: 0.66, 95% CI: 0.48, 0.91, P = 0.01).

**Table 2:** Multivariate logistic regression models of testosterone and CHF

	Model 1		Model 2		Model 3	
Total testosterone(ng/dl)	OR (95%CI)	P-value	OR (95%CI)	P-value	OR (95%CI)	P-value
Q1	Ref.		Ref.		Ref.	
Q2	0.60(0.38–0.93)	0.024	0.65(0.42, 1.00)	0.05	0.73(0.47, 1.14)	0.16
Q3	0.37(0.24–0.58)	< 0.001	0.44(0.28, 0.69)	< 0.001	0.54(0.33, 0.87)	0.01
Q4	0.29(0.19–0.47)	< 0.001	0.34(0.22, 0.54)	< 0.001	0.47(0.27, 0.80)	0.01
P-trend	< 0.0001		< 0.0001		< 0.001	

OR: odd ratio, CI: Confidence interval, CHF: congestive heart failure, BMI: Body mass index

Model 1: adjust for non

Model 2: adjust for age, ethnic

Model 3: adjust for age, ethnic, marital status, annual household income, education, smoke, alcohol consumption, BMI, diabetes, hypertension, Hyperlipidemia

**Table 3:** Multivariate logistic regression models of testosterone and CHF

	Model 1		Model 2		Model 3	
Total testosterone(ng/dl)	OR (95%CI)	P-value	OR (95%CI)	P-value	OR (95%CI)	P-value
< 300	Ref.		Ref.		Ref.	
≥ 300	0.46(0.33,0.63)	< 0.001	0.50(0.37, 0.69)	< 0.001	0.66(0.48, 0.91)	0.01

OR: odd ratio, CI: Confidence interval, CHF: congestive heart failure, BMI: Body mass index

Model 1: adjust for non

Model 2: adjust for age, ethnic

Model 3: adjust for age, ethnic, marital status, annual household income, education, smoke, alcohol consumption, BMI, diabetes, hypertension, Hyperlipidemia

**Fig. 3:**
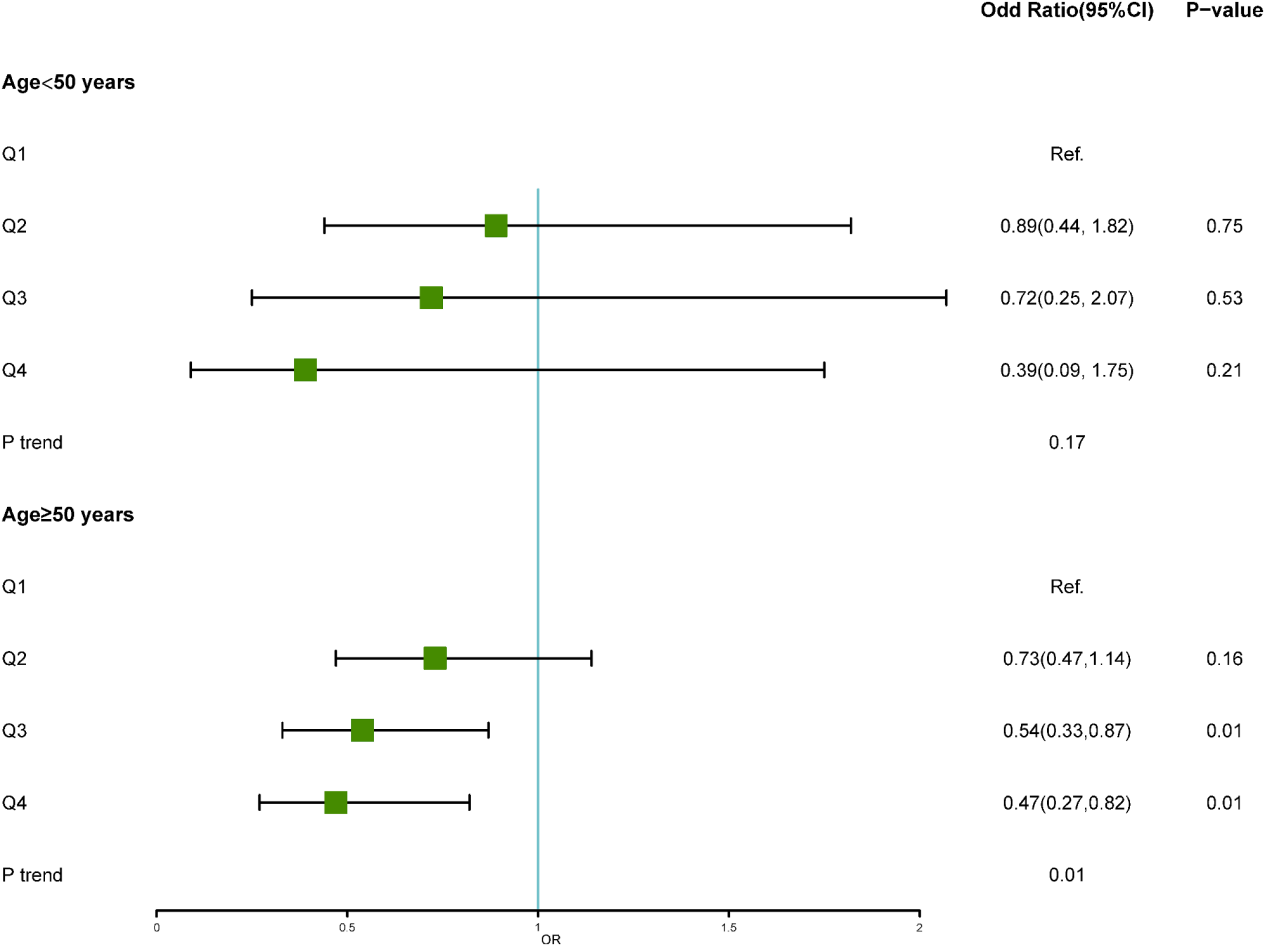
Association of serum testosterone with risk of congestive heart failure by age(< 50 years;≥50 years)

We performed subgroup analysis on the study population based on age and found a significant association between high serum testosterone levels and a reduced proportion of CHF only in the population over 50 years old (Q4 vs. Q1, odds ratio: 0.47, 95% confidence interval: 0.27–0.82, P = 0.01). However, no statistically significant correlation was observed between serum testosterone levels and CHF in the population under 50 years old (Fig. 3).

## Discussion

This study utilized data from the NHANES in the United States to investigate the association between serum testosterone levels and CHF in adult males. The results of the study demonstrated a linear relationship between low serum testosterone levels and CHF, with an increasing serum testosterone level being associated with a gradual reduction in the risk of CHF. After adjusting for other potential confounding factors, high serum testosterone levels remained significantly negatively correlated with CHF. Subgroup analysis revealed that this significant reduction in the proportion of CHF individual associated with high serum testosterone levels was only observed in the population aged 50 years and above.

Numerous studies have delved into the potential links between endogenous sex hormones, particularly serum testosterone, and cardiovascular disease (CVD). While CVD is a well-established global health concern, the association between serum testosterone and heart failure (HF) is still under investigation. The findings from existing studies, however, yielded inconsistent results, highlighting the necessity for further exploration in this area. In the context of men, an examination of a subset of participants in the Atherosclerosis Risk in Communities (ARIC) study did not uncover a statistically significant correlation between serum testosterone levels and the development of HF over a period of 12.8 years [8]. However, a separate and intriguing mendelian randomization study conducted using extensive data from the UK Biobank demonstrated a positive association between genetically predicted serum testosterone levels and the risk of incident HF [9]. These findings suggest a potential role of serum testosterone in influencing HF risk in men. Shifting our focus to women, the Multi-Ethnic Study of Atherosclerosis (MESA) did not observe any statistically significant associations between total serum testosterone, dehydroepiandrosterone (DHEA), sex hormone-binding globulin (SHBG) levels, and incident HF [10]. Nonetheless, total serum testosterone was identified as being positively associated with incident CVD and coronary heart disease (CHD) in women. Interestingly, DHEA exhibited an inverse relationship with HF with reduced ejection fraction (HFrEF), implying a potential protective effect against this specific type of HF. Furthermore, a similar mendelian randomization study conducted utilizing the UK Biobank data did not identify an association between genetically predicted serum testosterone levels and incident HF among women [9].

Serum testosterone was a crucial hormone involved in the development of heart failure, and it may have diverse effects on the cardiovascular system. Increased serum testosterone levels were associated with a decreased risk of heart failure, partially due to its protective effects on the vascular system [2]. Research suggested that serum testosterone could help prevent the development of conditions like atherosclerosis and thrombosis by reducing the release of inflammatory cytokines, improving endothelial function, stabilizing plaques, and reducing carotid intima-media thickness. Moreover, elevated serum testosterone levels in males were linked to lower blood pressure, reduced obesity, decreased left ventricular volume, and a lower risk of developing diabetes - all of which contributed to a decreased risk of heart failure. However, it’s important to note that serum testosterone could also have adverse effects on the cardiovascular system [11]. It possessed pro-inflammatory and vasoconstrictive properties, which may lead to increased blood pressure and other vascular abnormalities. Additionally, serum testosterone may promote the growth of muscle cells, potentially resulting in cardiac cell hypertrophy and myocardial thickening, which were commonly observed in heart failure with preserved ejection fraction. Postmenopausal women with elevated serum testosterone levels showed an increased risk of metabolic syndrome, endothelial dysfunction, atherosclerosis, and chronic heart disease. The associations in males, however, remained less clear. Understanding the cardiovascular effects of serum testosterone in specific populations was complex and currently inconclusive. Individual variations, coupled with other factors like metabolic status, hormone metabolism, and cardiovascular rehabilitation, likely influenced the effects of serum testosterone [12]. Therefore, while there appeared to be an association between increased serum testosterone levels and a lower risk of heart failure, further research was necessary to delineate the specific mechanisms and applicability of these findings.

An interesting finding in this study revealed an association between serum testosterone levels and the higher proportion of CHF exclusively among male individuals aged fifty and above. Conversely, no such association was observed in the population aged below fifty. The reason for this difference might be that the number of individuals in Q1 group in the population below 50 years old is relatively small compared to the other three groups, which results in a lack of statistically significant P value. We found that as the serum testosterone level increases, the obtained OR decreases, and the OR value of Q4 group in the population below 50 years old was also less than that in the population above 50 years old. It might be necessary to analyze a larger population of male individuals below 50 years old in the future to validate our research findings.

Low serum testosterone levels in men were associated with a significantly higher risk of CHF, potentially serving as an early warning indicator for this condition. Furthermore, there was a linear negative correlation between serum testosterone levels and the occurrence of CHF, indicating that higher serum testosterone levels were associated with a lower risk of developing CHF [13, 14]. This association was particularly significant in individuals aged 50 and older. These findings had important implications for clinical practice, as serum testosterone levels could serve as an adjunctive marker for predicting and diagnosing heart failure. For patients with low serum testosterone levels, further cardiovascular assessment and interventions may have been necessary to prevent the progression of heart failure. Additionally, maintaining appropriate serum testosterone levels may have helped prevent the onset of heart failure, especially in men aged 50 and above. However, to better understand the relationship between serum testosterone and heart failure, further research was needed to validate and explore the underlying mechanisms. Therefore, before incorporating these research findings into clinical decision-making, additional studies were required to evaluate the potential therapeutic effects of serum testosterone regulation on the development of heart failure.

One of the strengths of this study was the use of a large representative sample from NHANES, which gave our results a certain degree of generalizability and applicability to non-institutionalized civilian populations. Additionally, we implemented strict participant selection criteria, excluding individuals who were less likely to be associated with lower clinical serum testosterone decline in younger males and those with missing information on serum testosterone and CHF, thus increasing the reliability of the study. However, there were also some limitations to this study. Firstly, due to the cross-sectional design, it was challenging to determine causality. Secondly, the measurement of serum testosterone levels may have been influenced by various factors such as sampling time and laboratory techniques. Although we implemented strict controls during the measurement process, these factors may still have had some impact on the results. Thirdly, the diagnosis of CHF was based on a retrospective medical history questionnaire, which may have been subject to recall bias and information errors. Fourth, due to the relatively small number of individuals with low levels of serum testosterone in the population under 50 years old, we were unable to obtain statistically significant results in this age group. Lastly, the sample for this study primarily consisted of non-institutionalized civilian populations in the United States, which may have limited the generalizability of the results to other countries. For future research, it is recommended to employ longitudinal tracking study designs to determine whether there is a causal relationship between serum testosterone levels and the occurrence of CHF. Furthermore, further investigation into the biological mechanisms between serum testosterone levels and the onset of CHF would be beneficial in enhancing our understanding of their relationship.

## Conclusion

This study investigated the relationship between serum testosterone levels and CHF. The serum testosterone level was positively associated with CHF in adult males. Even after adjusting for other potential factors, individuals with higher serum testosterone levels still showed a trend of reduced CHF. Further analysis conducted on age groups revealed a significant association between high serum testosterone levels and reduced CHF in individuals aged 50 and above. However, no significant correlation between serum testosterone levels and CHF was observed in individuals below the age of 50. These findings have important implications for clinical decision-making and preventive measures. They provide new strategies for the prevention and treatment of CHF.

## Data Availability

Publicly available datasets were analyzed in this study. This. data can be found here: https://www.cdc.gov/nchs/nhanes.
